# Anatomically and functionally distinct locus coeruleus efferents mediate opposing effects on anxiety-like behavior

**DOI:** 10.1016/j.ynstr.2020.100284

**Published:** 2020-12-05

**Authors:** Olga Borodovitsyna, Brenna C. Duffy, Anthony E. Pickering, Daniel J. Chandler

**Affiliations:** a Department of Cell Biology and Neuroscience, Rowan University School of Osteopathic Medicine, 42 E. Laurel Road, Stratford, NJ, 08084, USA; b School of Physiology, Pharmacology & Neuroscience, University of Bristol, Biomedical Sciences Building, University Walk, Bristol, BS81TD, UK

**Keywords:** Locus coeruleus, Stress, Central nucleus of amygdala, Medial prefrontal cortex, Anxiety-like behavior, LC, locus coeruleus, CeA, central nucleus of the amygdala, mPFC, medial prefrontal cortex, NE, norepinephrine, TMT, 2,4,5-trimethylthiazole, EPM, elevated plus maze, OFT, open field test, PBS, phosphate buffered saline, aCSF, artificial cerebrospinal fluid, AHP, afterhyperpolarization, CRF, corticotropin releasing factor

## Abstract

The locus coeruleus (LC) is a critical node in the stress response, and its activation has been shown to promote hypervigilance and anxiety-like behavior. This noradrenergic nucleus has historically been considered homogeneous with highly divergent neurons that operate en masse to collectively affect central nervous system function and behavioral state. However, in recent years, LC has been identified as a heterogeneous structure whose neurons innervate discrete terminal fields and contribute to distinct aspects of behavior. We have previously shown that in late adolescent male rats, an acute traumatic stressor, simultaneous physical restraint and exposure to predator odor, preferentially induces c-Fos expression in a subset of dorsal LC neurons and persistently increases anxiety-like behavior. To investigate how these neurons respond to and contribute to the behavioral response to stress, we used a combination of retrograde tracing, whole-cell patch clamp electrophysiology, and chemogenetics. Here we show that LC neurons innervating the central nucleus of the amygdala (CeA) and medial prefrontal cortex (mPFC) undergo distinct electrophysiological changes in response to stressor exposure and have opposing roles in mediating anxiety-like behavior. While neurons innervating CeA become more excitable in response to stress and promote anxiety-like behavior, those innervating mPFC become less excitable and appear to promote exploration. These findings show that LC neurons innervating distinct terminal fields have unique physiological responses to particular stimuli. Furthermore, these observations advance the understanding of the LC as a complex and heterogeneous structure whose neurons maintain unique roles in various forms of behavior.

## Highlights

• Locus coeruleus-central amygdala projections are hyperactive one week after stress.
• Locus coeruleus-prefrontal cortex projections are hypoactive one week after stress.
• Chemogenetic manipulation of each pathway distinctly affects anxiety-like behavior.

## 1 Introduction

The locus coeruleus (LC) is the largest cluster of noradrenergic neurons in the brain and has historically been viewed as a largely homogeneous structure. Through a network of highly ramified axons, it has been thought to innervate vast expanses of anatomically and functionally disparate terminal fields (Morrison et al., 1978; Loughlin et al., 1982; Aston-Jones et al., 1986). Accordingly, LC has been recognized to contribute to myriad central nervous system functions, including sensory signal processing (Waterhouse et al., 1998; Bouret and Sara 2002), spinal nociception (Hirschberg et al., 2017), sleep/wake cycles (Aston-Jones and Bloom 1981; Berridge et al., 2012), cognition (Arnsten 1998; Sara and Bouret 2012), feeding (Sciolino et al., 2019), and affect (McCall et al., 2015). The role of LC in the stress response has also been extensively studied. Stress increases LC tonic discharge rates which correlate highly with forebrain norepinephrine (NE) release and behavioral indices of arousal (Berridge et al., 2012) While optogenetic stimulation of LC has been shown to promote aversion and anxiety-like behavior (McCall et al., 2015), some studies have yielded conflicting results on these behaviors. While genetic knockout of dopamine-β-hydroxylase, the NE synthetic enzyme, reduces neophobia (Lustberg et al., 2020), depletion of forebrain NE by injection of the neurotoxin DSP-4 has been shown to increase it (Harro et al., 1995). However, a lower dose of DSP-4 that reduces cortical but not hypothalamic NE instead produces an anxiolytic effect (Lapiz et al., 2001), while selective noradrenergic denervation of the basolateral amygdala with the neurotoxin 6-OHDA increases anxiety-like behavior (Ferrazzo et al., 2019).

These conflicting reports suggest that the role of LC in mediating anxiety-like behavior is more nuanced and depends on the activation of specific circuits. Indeed, several recent studies have begun to reveal a more complicated view of LC organization and function. LC neurons are more restricted in their efferent anatomy than originally recognized, with subsets of LC neurons projecting to specific terminal fields varying in their biological properties and contributing to distinct aspects of behavior (Hirschberg et al., 2017; Uematsu et al., 2017; Chandler et al., 2019). Furthermore, while it was originally thought that LC neurons operate in a coupled mode which allows them to fire synchronously*,* recent observations suggest that they operate independently of one another (Totah et al. 2018, 2019). Such heterogeneity makes it unclear if all LC neurons contribute to anxiety-like behavior in the same way, or if various subsets of neurons encode distinct aspects of anxiety-like behavior. In a previous study, we found that acute traumatic stress induced c-Fos expression preferentially in a subset of dorsal LC neurons (Borodovitsyna et al., 2018). The medial prefrontal cortex (mPFC) is innervated by dorsal LC neurons (Loughlin et al., 1982; Loughlin et al., 1986; Hirschberg et al., 2017) which release NE in this region during stress (Finlay et al., 1995). The central nucleus of the amygdala (CeA) also receives input from dorsal LC (Mason and Fibiger 1979; Jones and Yang 1985; Schwarz et al., 2015) and contributes directly to anxiety-like behavior (Gilpin et al., 2015; Gu et al., 2020). We therefore sought to determine if the LC projection to these regions constitute parallel stress-responsive circuits that contribute to the formation of anxiety-like behavior in response to stressor exposure.

To interrogate the function of these cells and their relationship to the stress response and anxiety-like behavior, we used a combination of retrograde tracing, whole-cell patch clamp electrophysiology, and circuit-specific chemogenetics. Our results show that LC cells innervating mPFC and CeA undergo opposing adaptations in response to acute traumatic stress, and their activity promotes opposing effects on anxiety-like and motor behavior. These findings provide important new evidence for a modular LC whose neurons are restricted in their efferent anatomy and unique in their function.

## 2 Materials and methods

### 2.1 Subjects

Male Sprague Dawley rats (Taconic Farms) were housed two to three per cage on a 12 h reverse light schedule (lights on at 9:00pm) with access to standard rat chow and water *ad libitum*. Animal protocols were approved by the Rowan University Institutional Animal Care and Use Committee and were conducted in accordance with National Institutes of Health *Guide for the Care and Use of Laboratory Animals.*

### 2.2 Surgery

Surgical procedures were performed according to a standard protocol as we have described previously (Chandler et al., 2014). Briefly, rats were deeply anesthetized through isoflurane inhalation (4% induction, 1–2% maintenance) and placed in a stereotaxic frame. Rats for chemogenetic studies were approximately five weeks of age at the time of surgery, while rats for retrograde tracing were approximately seven weeks of age so that all rats would be approximately eight weeks of age (late adolescents/young adults) at the time of stressor exposure. This allowed additional survival time for virally transduced rats to permit transgene expression. Coordinates for injections were as follows: mPFC from bregma: AP = +3.2 mm; ML = ±1.8 mm, DV = −3.9 mm @ 15° from vertical in the coronal plane; CeA from bregma: AP = 2.0 mm; ML = ±4.0 mm; DV = −7.0 mm from the dura; LC from lambda: AP = −1.2 mm; ML = ±2.7 mm; DV = −6.6 mm @ 15° from vertical in the coronal plane. For double retrograde tracing experiments, all animals (n = 14) received a 0.3 μl injection of one 10 kDa dextran (fluorescein or rhodamine conjugated, 2% in 0.1 M PBS, Invitrogen) into left mPFC and another 0.3 μl injection of the other fluorescent 10 kDa dextran into left CeA. Injections of fluorescein and rhodamine were counterbalanced between surgeries. For single retrograde tracer experiments for electrophysiology studies, rats received a single 0.3 μl injection of 10 kDa rhodamine conjugated dextran (2% in 0.1 M PBS, Invitrogen) in mPFC (at the juncture of prelimbic and infralimbic cortices, n = 8) or CeA (n = 11). For chemogenetic experiments, animals received a single 0.3 μL injection of CAV2-PRS-CreV5 (1.4 x 10^12^IU/mL; provided by AEP; available from Institut Génétique Moléculaire de Montpellier) into mPFC (n = 16 total) or CeA (n = 16 total) bilaterally, and a single 0.3 μL injection of AAV2-hSyn-DIO-hM3Dq-mCherry (n = 4 of 16 for each forebrain injection site; 5 x 10^12^ vg/mL), or AAV2-hSyn-DIO-hM4Di-mCherry (n = 4 of 16 for each forebrain injection site; 6 x 10^12^ vg/mL), or AAV2-hSyn-DIO-mCherry (n = 8 for each forebrain injection site, 4 x 10^12^ vg/mL) into LC, bilaterally. CAV2-PRS-CreV5 is retrogradely transported and uses a 240-base pair synthetic promoter sequence to selectively drive expression of Cre recombinase and a V5 tag in noradrenergic neurons due to their expression of the Phox2 transcription factor. This vector has previously been validated and used to transduce LC neurons (Hayat et al., 2020) and we have previously used a similar CAV2-Cre construct to retrogradely transduce LC neurons in mice (Plummer et al., 2020). All injections were performed using a 1.0 μL Hamilton Neuros syringe mounted in a World Precision Instruments stereotax-mounted injection pump at a flow rate of 50 nL/min. Syringes remained in place for 10 min before removal. Craniotomies were filled with sterile bone wax, and the incision was closed with wound clips. Following surgery, rats underwent a one week (retrograde tracer infusions) or three week (viral infusions) recovery to permit tracer transport and transgene expression. All AAVs were gifts from Bryan Roth (AddGene viral preps 44,361, 44,362, and 50,459). Injection sites were confirmed histologically after the completion of all experiments.

### 2.3 Chemogenetics

Thirty minutes prior to stressor exposure or control conditions, rats received an intraperitoneal injection of clozapine-N-oxide (CNO, 2.5 mg/kg, Tocris) dissolved in sterile saline. Rats that received AAV2-hSyn-DIO-hM3Dq-mCherry underwent control conditions, and rats that received AAV2-hSyn-DIO-hM4Di-mCherry underwent stress conditions. Vector/fluorophore control rats received AAV2-hSyn-DIO-mCherry in LC and intraperitoneal injections of 2.5 mg/kg CNO 30 min prior to control or stress conditions. In this way, there were four possible conditions to assess how LC→mPFC and LC→CeA neurons each contribute to anxiety-like behavior: activation + control, inhibition + stress, no change in activity + control, no change in activity + stress. The effect of manipulation of each pathway was assessed by one-way ANOVA.

### 2.4 Stressor exposure

Stressor exposure occurred as we have previously described (Borodovitsyna et al., 2018). Rats were handled by the experimenter for 5–10 min per day during recovery prior to control or stress conditions to habituate them to experimental handling. Rats were also habituated to a plastic chamber where stress or control conditions took place. Stress and control conditions, as well as behavioral testing, took place in a dimly lit room. Acute stress was induced by placing rats in a rodent restrainer (Harvard Apparatus) for 15 min which was placed inside of a sealed anesthesia induction chamber connected by silicone tubing to an aquarium pump. A small plastic tube was positioned in-line with the tubing. A 2.5 cm × 2.5 cm piece of filter paper was placed inside of the tube and saturated with 100 μL predator odor (2,4,5-trimethylthiazole, TMT, Sigma-Aldrich). Odor delivery was achieved by turning on the aquarium pump so that the air forced through the tubing carried the odor into the airtight odor exposure chamber.

### 2.5 Elevated plus maze

Immediately after exposure to stress or control conditions, rats were placed in the center of an elevated plus maze (EPM) in a dark room. The EPM consisted of a plus shaped black plexiglass apparatus elevated 76 cm off the ground with two sets of opposing arms (each arm = 40 cm in length) meeting in a central 10 cm × 10 cm area. Two opposing arms have vertical walls extending 30 cm from the floor of the maze, while the other two arms do not have walls. Rats were allowed to explore the maze for 10 min. Their activity was filmed with an infrared camera situated above the maze connected to a Lenovo ThinkCentre M700 PC. At the conclusion of each test, rats were either returned to their home cage for a week, or sacrificed for electrophysiological recordings. The maze was cleaned with 10% bleach between each test. Open arm time, time freezing, time mobile, and average speed were scored using AnyMaze behavioral tracking software (Stoelting, RRID SCR_014289). The onset of freezing episodes was defined by a period of 1s without motion, and were terminated when motion was again detected. In some cases as indicated in figure legends, open arm time, time freezing, and average speed were square root transformed to satisfy normality and homogeneity of variance requirements for parametric statistical testing.

### 2.6 Open field test

One week after testing in the EPM, rats were placed in the center of an open field test (OFT) in a dark room. The OFT consisted of a 90 cm × 90 cm x 30 cm black plexiglass box. Rats were allowed to explore the apparatus for 10 min, during which their activity was filmed with an infrared camera situated above the maze connected to a Lenovo ThinkCentre M700 PC. At the conclusion of the test, rats were sacrificed for either electrophysiological recordings or histology. The apparatus was cleaned with 10% bleach between each test. Center time and time freezing were scored using AnyMaze behavioral tracking software (Stoelting, RRID SCR_014289). The onset of freezing episodes was defined by a period of 1s without motion, and were terminated when motion was again detected. EPM was used immediately after stressor exposure and OFT was used one week after to eliminate the possibility of habituation to any one test confounding anxiety-like behavior, however, we have previously shown that stress-induced anxiety-like behavior in these two tests is highly correlated (Borodovitsyna et al., 2018). In some cases as indicated in figure legends, time freezing in the OFT was square root transformed to satisfy normality and homogeneity of variance requirements for parametric statistical testing.

### 2.7 Immunohistochemistry

Rats were transcardially perfused with 300 mL 0.9% NaCl and 300 mL 4% paraformaldehyde in 0.1 M phosphate buffer. Brains were extracted, post-fixed, cryoprotected, and sliced in the coronal plane to generate a 1:6 series of sections containing LC. Free-floating sections were washed in 0.1 M phosphate buffered saline (PBS), blocked in 4% normal donkey serum, incubated in mouse anti-dopamine β hydroxylase (1:1000, Chemicon MAB308, RRID AB_2245740) and rabbit anti-c-Fos (1:1000, Santa Cruz sc-52, RRID AB_2106783) @ 4 °C for 48 h, washed again in 0.1 M PBS, incubated in AlexaFluor 647 donkey anti-mouse (1:1000, Invitrogen, RRID AB_162542) and AlexaFluor 350 donkey anti-rabbit (1:1000, Invitrogen, RRID AB_2534015) secondary antibodies, washed in 0.1 M PBS, and mounted on gelatin-coated glass slides. Fluorescent photomicrographs were generated with on a Leica DMR fluorescence microscope equipped with a QImaging Retiga R6 camera using QImaging Ocular software. Images were processed in ImageJ and numbers of retrogradely labeled neurons and c-Fos positive nuclei were manually counted. The number of c-Fos positive nuclei was logarithm transformed to satisfy normality and homogeneity of variance requirements for parametric statistical testing. Logarithm transformation was used in this case because square root transformation failed to normalize the data sets.

### 2.8 Brain slice preparation

Rats were deeply anesthetized with an intraperitoneal injection of Euthasol (100 mg/kg, Virbac) and transcardially perfused with 60 mL ice cold oxygenated artificial cerebrospinal fluid (aCSF) of the following composition, in mM: NaCl 126, KCl 2.5, CaCl_2_ 2.4, NaH_2_PO_4_ 1.2, MgCl_2_ 1.3, NaHCO_3_ 25, D-glucose 11. Rats were then rapidly decapitated and the skull was removed so that gross coronal cuts could be made at the level of the medulla and the pineal gland; the resulting block of brain tissue was then extracted from the skull and transferred to 30 mL of ice cold oxygenated sucrose-aCSF of the following composition, in mM: sucrose 58.4, NaCl 85, KCl 2.5, CaCl_2_ 2.4, NaH_2_PO_4_ 1.2, MgCl_2_ 1.3, NaHCO_3_ 25. The brain remained in the sucrose-aCSF for 1–2 min after which it was transferred to a piece of filter paper saturated with ice cold oxygenated sucrose aCSF, and the lateral edges of the brain were trimmed off. The dorsal aspect of the brain was then glued to the stage of a Compresstome VF-300-0Z tissue slicer, embedded in agarose, submerged in ice cold oxygenated sucrose aCSF and 200 μM thick horizontal sections were cut at a speed of 0.1 mm/s with an amplitude of 1.0 mm. Sections containing LC (typically, 3 to 4 per animal) were transferred to a holding incubator containing ~300 mL aCSF continuously bubbled with 95% O_2_/5% CO_2_ maintained at 35.5 °C and supported by nylon mesh for 1 h. After 1 h, the holding incubator was maintained at room temperature.

### 2.9 Electrophysiological recordings

Brain slices were individually transferred to a recording chamber which was continuously superfused at 1.5–2 mL/min with oxygenated aCSF maintained at 37 °C by a Warner Instrument Corporation in-line heater (model 60–01013). LC was visualized as a semi-translucent crescent-shaped region located lateral to the fourth ventricle at 5X magnification using an Olympus BX51WI fixed-stage upright microscope with differential interference contrast and an infrared filter. Individual LC neurons were visualized with a 40X immersion lens and QImaging Rolera Bolt camera connected to a Lenovo ThinkCentre M700 desktop computer using QCapture Pro software. An X-Cite 120 LED Boost system was used to illuminate retrogradely labeled cells in red fluorescence. Only a single red tracer was injected in either CeA or mPFC in these studies because fluorescent green tracer was found to photobleach too quickly in living tissue to permit patching of targeted neurons. Labeled neurons were found primarily in the dorsal half of LC but those present in more ventral sections were also recorded when possible. Neurons were approached with patch electrodes (resistance = 5–10 MΩ) controlled with Sutter MPC-200 manipulators. Electrodes were filled with intracellular solution of the following composition, in mM: KCl 20, K-gluconate 120, MgCl_2_ 2, EGTA 0.2, HEPES 10, Na_2_ATP 2. After a GΩ seal was established between the pipette and neuronal membrane, the membrane was ruptured, and neurons were allowed to equilibrate for 2–3 min prior to data acquisition. Whole-cell recordings were made with a MultiClamp 700 B amplifier, Digidata 1550 B digitizer equipped with two HumSilencer channels, and ClampEx 10.6 software. Electrophysiological data were analyzed using a two-factor design (treatment: control vs stress, and terminal field: mPFC vs CeA). To assess membrane properties in current clamp mode, spontaneous activity was recorded for 60s without any input and the average firing rate was calculated. They were then subject to a series of increasing current steps from −250pA to 300 pA with 50 pA intervals between sweeps, and the input resistance and number of action potentials fired in response to each level of current was determined. Spontaneous firing rate was square root transformed to satisfy normality and homogeneity of variance requirements for parametric statistical testing. Activation gap was computed as the voltage difference between resting membrane potential and threshold. Afterhyperpolarization (AHP) was computed as the voltage difference between action potential threshold and the most hyperpolarized potential that occurred after an action potential. Electrophysiological data were analyzed with Molecular Devices ClampFit 10.6 software.

### 2.10 Experimental design and statistical analysis

Statistical analyses were performed with GraphPad Prism version 8.4.1. All data sets were tested for normality and homogeneity of variance. Those that satisfied both of these requirements underwent parametric testing as indicated in the text. Due to data sets being on the interval and ratio scales, and to preserve statistical power, those that did not meet normality or homogeneity of variance requirements were transformed as indicated in the text and then analyzed using parametric tests. Data are presented as mean ± SEM for normally distributed data sets and median with interquartile range for non-normally distributed data sets as indicated in figure legends.

## 3 Results

### *3.1* *Acute stressor exposure drives greater c-Fos expression in LC*→*CeA than LC→mPFC projection neurons*

**Fig. 1:**
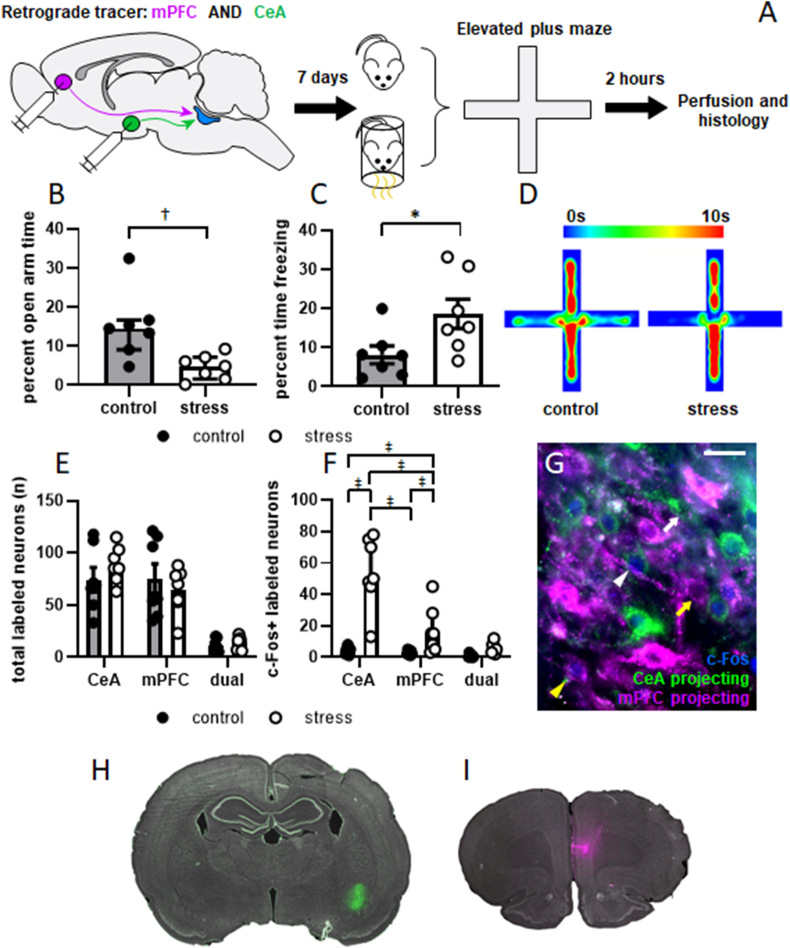
**Acute stressor exposure increases anxiety-like behavior and c-Fos expression in LC neurons projecting to CeA.** The experimental timeline is shown in A. Animals underwent a surgical procedure to inject two distinct retrograde tracers in mPFC (red; shown in magenta) and CeA (green). Seven days later, they underwent stressor exposure (n = 7) or control conditions (n = 7). Behavior was then assessed in the EPM, and 2 h later rats were sacrificed for immunofluorescent detection of c-Fos in LC. Time spent in the open arms was square-root transformed to satisfy requirements for parametric statistical testing and was significantly decreased in stressed rats (gray bars with filled circles) relative to control rats (white bars with open circles; B; shown as median with interquartile range). Stressed rats also spent more time freezing than control rats (C). Mean heat maps for behavior in the EPM are shown in D. Injections of retrograde tracers resulted in similar numbers of labeled cells projecting to mPFC and CeA regardless of treatment. The number of double labeled cells projecting to both regions (dual) was small (E). The number of c-Fos positive nuclei was logarithm transformed to satisfy requirements for parametric testing, and was found to significantly increase in both the LC→CeA and LC→mPFC projections in response to stressor exposure. However, the number of c-Fos positive nuclei was significantly greater in the LC→CeA projection than LC→mPFC projection in stressed animals (F; shown as median with interquartile range). A representative photomicrograph of labeled neurons in the dorsal third of LC is shown in G. LC→mPFC neurons are magenta, LC→CeA neurons are green, and c-Fos is shown in blue. White arrowhead and arrow indicate examples of c-Fos+ and c-Fos- LC→CeA neurons, respectively. Yellow arrowhead and arrow indicate examples of c-Fos+ and c-Fos- LC→mPFC neurons, respectively. Scale bar = 25 μm. Representative images of injection of tracers into CeA and mPFC are shown H and I, respectively. *: p < 0.05. †: p < 0.05 after square root transformation. ‡: p < 0.05 after logarithm transformation. (For interpretation of the references to colour in this figure legend, the reader is referred to the Web version of this article.)

Previous observations from our laboratory show that a single episode of combined physical restraint and predator odor exposure induces c-Fos expression in a dorsally located subset of LC neurons (Borodovitsyna et al., 2018). To determine if these cells belonged to part of a stress-related circuit, rats (n = 14) underwent surgery to inject two distinctly labeled retrograde tracers into mPFC and CeA. One week later, rats underwent stressor exposure or control conditions and were then tested in the EPM (Fig. 1A). Percent open arm time was square root transformed to meet requirements for parametric statistical testing and an unpaired *t*-test showed that stress significantly decreased this measure (t = 3.388, p = 0.0054; Fig. 1B). Percent time freezing in the EPM was also significantly increased by stressor exposure (t = 2.385, p = 0.0344; Fig. 1C). Mean heat maps for activity in the EPM are shown in Fig. 1D. Two hours later, rats were sacrificed and their brains were processed for c-Fos immunofluorescent staining. Consistent with our previous findings (Chandler et al., 2014), LC→mPFC and LC→CeA projection neurons were largely anatomically independent of one another: on average, 7.59% ± 0.9% of labeled cells contained both tacers (7.1% ± 0.8%, of total labeled cells in control animals and 8.1% ± 1.1% of total labeled cells in stressed animals). Of these, 7.5% ± 3.6% expressed c-Fos in control animals, and 46.44% ± 8.8% expressed c-Fos in stressed animals. Because the number of cells within this population was so small, it was not subject to any statistical analysis. A two-way ANOVA showed that the number of singly retrogradely labeled LC neurons was unaffected by terminal field (mPFC or CeA; F [1,24] = 1.247, p = 0.2753), treatment (control or stress; F [1,24] = 0.02189, p = 0.8836), or the terminal field × treatment interaction (F [1,24] = 1.498, p = 0.2328; Fig. 1E). The number of c-Fos positive neurons within each population was found to be significantly affected by treatment (F [1,24] = 57.67, p < 0.0001), terminal field (F [1,24] = 10.54, p = 0.0034) and the terminal field × treatment interaction (F [1,24] = 4.451, p = 0.0455; Fig. 1F). Post hoc tests with Bonferroni corrections applied showed that stressor exposure significantly increased c-Fos expression in both the LC→CeA projection neurons (p < 0.0001) and the LC→mPFC projection neurons (p = 0.0037). However, c-Fos expression was significantly greater in the LC→CeA projection in stressed rats than the LC→mPFC projection of stressed rats (p = 0.0047). A representative image showing c-Fos labeling within each population is shown in Fig. 1G. Consistent with our previous findings, c-Fos positive nuclei were generally more dense in the dorsal aspect of LC. Retrogradely labeled neurons projecting to both mPFC and CeA spanned the dorsoventral axis of LC, although they were also more densely present in the dorsal aspect than ventral. This is consistent with prior reports of a rough efferent topography within LC (Loughlin et al., 1982; Hirschberg et al., 2017). Representative images of tracer injections in CeA and mPFC are shown in Fig. 1H and I, respectively. These findings show that despite activation of both of these pathways by stress, the LC projection to CeA is activated to a greater degree than the projection to mPFC.

### *3.2* *LC*→*mPFC and LC*→*CeA projection cells undergo opposing adaptations one week after stressor exposure*

**Fig. 2:**
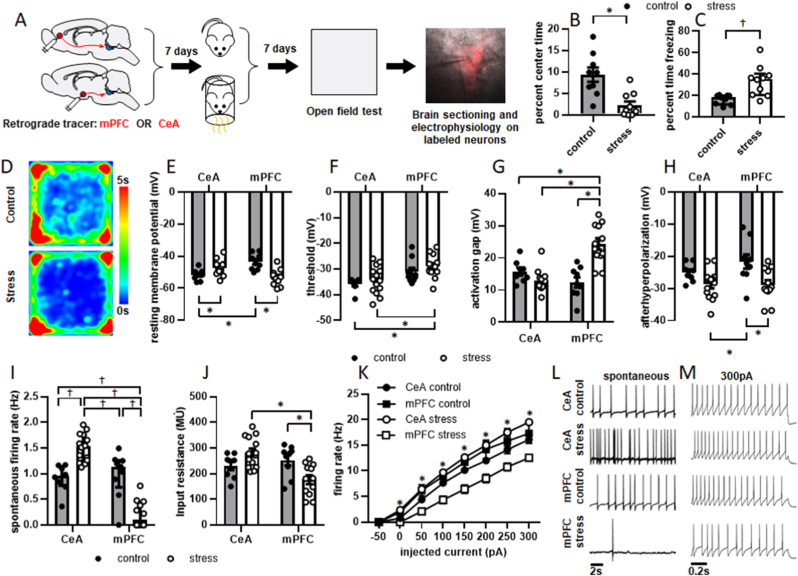
**Acute stressor exposure increases anxiety-like behavior and alters LC neuronal membrane properties one week later.** The experimental timeline is shown in A. Animals underwent a surgical procedure to inject a red fluorescent retrograde tracer into mPFC or CeA. Seven days later, they underwent stressor exposure (n = 10) or control conditions (n = 9). One week later, anxiety-like behavior was assessed in the OFT. Rats were then sacrificed for whole-cell recordings of retrogradely labeled LC neurons. Stressor-exposed rats spent significantly less time in the center of the OFT (B). Time freezing was square root transformed to satisfy requirements for parametric statistical testing and found to significantly increase in response to stress (C; shown as median with interquartile range). Mean heat maps for behavior in the EPM are shown in D. LC→CeA neurons from stressed animals (n = 14 cells from 6 rats) were significantly more depolarized than LC→CeA neurons from control animals (n = 9 cells from 5 rats), while and LC→mPFC neurons from stressed rats (n = 12 cells from 4 rats) wre significantly more depolarized than LC→mPFC neurons from control animals (n = 9 cells from 4 rats). LC→mPFC neurons from control rats were also significantly more depolarized than LC→CeA neurons in control animals (E). Action potential threshold was also significantly more depolarized in LC→mPFC cells from stressed animals than in LC→CeA cells in both control and stressed animals (F). Activation gap, or the voltage required to reach threshold from rest, was significantly increased in LC→mPFC cells by stressor exposure. It was also significantly higher in LC→mPFC neurons from stressed animals than in LC→CeA neurons from either control or stressed animals (G). Afterhyperpolarization was significantly increased in LC→mPFC neurons by stressor exposure, and was significantly larger in LC→CeA cells from stressed animals than in LC→mPFC cells from control animals (H). Spontaneous firing rates were square root transformed to meet requirements for parametric statistical testing. Stress significantly increased the spontaneous firing rate of LC→CeA neurons, but decreased it in LC→mPFC projection cells (I; shown as median with interquartile range). Input resistance was significantly decreased by stressor exposure in the LC→mPFC cells, and was significantly greater in LC→CeA cells than LC→mPFC cells within the stress group (J). Stressor exposure and terminal field both significantly affected neuronal responsiveness to all levels of current injection (K). A summary of significant post hoc tests for results in (K) is shown in Table 1. Representative traces of spontaneous and evoked firing in response to 300 pA current injection are shown in L and M, respectively. *: p < 0.05. †: p < 0.05 after square root transformation. (For interpretation of the references to colour in this figure legend, the reader is referred to the Web version of this article.)

Based on the observation that LC cells innervating mPFC and CeA express c-Fos at different levels in response to stressor exposure, we sought to identify if they respond to stressor exposure differently. We have previously shown that acute stressor exposure persistently alters the electrophysiological parameters of patched LC neurons selected at random (Borodovitsyna et al., 2018). However, LC physiological properties vary according to terminal field (Chandler et al., 2014; Li et al., 2016), and the variability within each measured parameter was substantially larger within the stress group than the control group. This suggested that LC cells innervating distinct terminal fields might respond to stress differently. To investigate this, animals underwent a surgical procedure to inject a red fluorescent retrograde tracer into either mPFC or CeA. One week later, rats underwent control or stress conditions and were then returned to their home cages. One week later, rats underwent testing in the OFT and were then sacrificed for electrophysiological recordings (Fig. 2A). An unpaired *t*-test showed that stressor exposure significantly decreased percent time spent in the center of the OFT (t = 3.925, p = 0.0011; Fig. 2B) and increased percent time freezing (t = 3.511, p = 0.0027; Fig. 2C). Mean heat maps for behavior in the OFT are shown in Fig. 2D.

Locus coeruleus membrane properties were in general agreement with prior reports (Williams et al., 1984; Ishimatsu and Williams 1996; Jedema and Grace 2004), with 16 of 17 recorded cells from control animals showing spontaneous activity. A two-way ANOVA failed to detect significant main effects of treatment (F [1,39] = 3.386, p = 0.0734) or terminal field (F [1,39] = 0.5177, p = 0.4761) on resting membrane potential (Fig. 2E), however, the treatment x terminal field interaction was significant (F [1,39] = 24.68, p < 0.0001). Bonferroni-adjusted post-hoc tests revealed that resting membrane potential was significantly more depolarized in LC→mPFC cells than LC→CeA cells in control rats (p = 0.0045). Stress also caused a significant depolarization of resting membrane potential in LC→CeA projection cells (p = 0.0101), but a significant hyperpolarization in LC→mPFC projection cells (p = 0.0001). This indicates that stressor exposure reduces the likelihood that LC→mPFC cells will fire action potentials upon stimulation, while LC→CeA cells are more likely to do so.

A two-way ANOVA failed to detect significant main effects of treatment (F [1,39] = 1.696, p = 0.2004) or treatment x terminal field interaction (F [1,39] = 0.039, p = 0.8443) on action potential threshold (Fig. 2F), but there was a significant main effect of terminal field (F [1,39] = 12.12, p = 0.0012). Bonferroni-adjusted post-hoc tests revealed that threshold was significantly more hyperpolarized and thus closer to rest in LC→CeA projection cells than LC→mPFC projection cells in the stressed rats (p = 0.0345). Additionally, threshold was significantly more hyperpolarized in the LC→CeA projection cells in control rats than LC→mPFC cells in stressed rats (p = 0.0133). This indicates that stressor exposure increases the likelihood that LC→CeA cells will reach threshold and generate action potentials upon stimulation relative to both LC→CeA cells from control animals and LC→mPFC cells from stressed animals.

A two-way ANOVA revealed significant main effects of both treatment (F [1,39] = 10.11, p = 0.0029) and terminal field (F [1,39] = 7.977, p = 0.0074) as well as a significant treatment x terminal field interaction effect (F [1,39] = 27.03, p < 0.0001) on activation gap (Fig. 2G). Bonferroni-adjusted post-hoc tests revealed that significantly less voltage was needed to reach threshold from rest in LC→CeA cells than LC→mPFC cells among the stressed rats (p < 0.0001). Additionally, activation gap was significantly greater in the LC→mPFC projection cells in stressed rats than controls (p < 0.0001). Activation gap was also significantly greater in LC→mPFC projection cells from stressed rats than in LC→CeA cells in control rats (p = 0.0012). This indicates that stressor exposure increases the amount of stimulation that LC→mPFC cells require to reach threshold from rest. Additionally, among stressed rats, LC→CeA cells also require significantly less stimulation than LC→mPFC cells to reach threshold from rest.

A two-way ANOVA revealed a significant main effect treatment (F [1,39] = 12.5, p = 0.0011) on afterhyperpolarization (AHP; Fig. 2H), but neither main effect of terminal field (F [1,39] = 1.099, p = 0.301) nor treatment x terminal field interaction (F [1,39] = 1.277, p < 0.2653) were found to be significant. Bonferroni-adjusted post-hoc tests revealed that stress significantly increased AHP in the LC→mPFC projection cells relative to controls (p = 0.0122), and that AHP was significantly smaller in LC→mPFC cells from control rats than in LC→CeA cells from stressed rats (p = 0.0107). This indicates that LC→mPFC cells are more potently inhibited following action potential generation and thus require more time to return to rest in stressed animals than controls.

A two-way ANOVA failed to detect a significant main effect of treatment (F [1,39] = 0.7756, p = 0.3839) on spontaneous firing rate (Fig. 2I); however, it was significantly affected by LC neuronal terminal field (F [1,39] = 39.02, p < 0.0001) and the treatment x terminal field interaction (F [1,39] = 48.73, p < 0.0001). Bonferroni-adjusted post-hoc tests revealed that stress significantly increased the firing rate in the LC→CeA cells relative to controls (p = 0.0007) but decreased it in the LC→mPFC cells relative to controls (p < 0.0001). Notably, half of the LC→mPFC neurons from stressed rats showed no spontaneous firing. This indicates that LC→mPFC cells become suppressed, but LC→CeA cells become hyperactive in response to stressor exposure.

A two-way ANOVA failed to detect a significant main effect of treatment (F [1,39] = 0.4644, p = 0.4996) on input resistance (Fig. 2J); however, this measure was significantly affected by LC neuronal terminal field (F [1,39] = 5.348, p = 0.0261) and the treatment x terminal field interaction (F [1,39] = 12.19, p = 0.012). Bonferroni-adjusted post-hoc tests revealed that stress significantly decreased input resistance in the LC→mPFC projection (p = 0.0314) but not the LC→CeA projection (p = 0.3726). The input resistance of the LC→CeA cells was also significantly higher than the LC→mPFC projection cells among the stressed rats (p = 0.0003). These observations suggest that there is increased channel conductance in the LC→mPFC population in response to stressor exposure.

To further determine the effects of terminal field and stressor exposure on LC neuronal excitability, a series of 1s long current steps (−50 → 300 pA, 50 pA intervals between sweeps) was injected into cells, and the number of action potentials generated in response to each was recorded (Fig. 2K). A different two-way ANOVA was used to assess the effects of terminal field and treatment on action potential generation at each level of current injection rather than including injected current as a third variable, as it is known that LC firing frequency increases linearly with injected current. There was no significant main effect of treatment at any level (p > 0.05 in all cases). There was a significant main effect of terminal field on firing rate at all levels above 50 pA, and a significant terminal field × treatment interaction at all levels above −50pA. Individual F and p values for all levels of injected current, as well as Bonferroni-adjusted p values for relevant significant post hoc comparisons, are shown in Table 1. This set of experiments showed that at all levels of current injection, LC→mPFC neuronal excitability is significantly decreased by stress. Additionally, within stressed animals, LC→CeA neurons are significantly more excitable than LC→mPFC neurons at all levels of current injection. Representative traces of spontaneous and evoked activity within each population are shown in Fig. 2L and M, respectively. Collectively, these findings suggest that the activity, excitability, and likelihood of firing in response to afferent stimulation are suppressed by stress in LC→mPFC neurons, but enhanced in LC→CeA neurons.

**Table 1:** Summary of significant effects of stressor exposure on LC firing rate in response to increasing levels of current injection.

	terminal field		terminal field x treatment		LC→mPFC: control vs stress	LC→CeA: control vs stress	stress: LC→mPFC vs LC→CeA
pA	F (1,39)	p	F (1,39)	p	Bonferroni-adjusted p	Bonferroni-adjusted p	Bonferroni-adjusted p
0	2.985	ns	24.58	<0.0001	0.003	0.0157	<0.0001
50	3.997	ns	24.56	<0.0001	0.0002	ns	<0.0001
100	8.795	0.0051	22.91	<0.0001	0.0002	ns	<0.0001
150	7.372	0.0098	20.27	<0.0001	0.0006	ns	<0.0001
200	4.321	0.0443	17.96	0.0001	0.002	ns	<0.0001
250	5.683	0.0221	15.44	0.0003	0.0113	ns	<0.0001
300	7.717	0.0084	7.717	0.0084	0.0132	ns	<0.0001

### *3.3* *LC*→*CeA neurons are necessary and sufficient for the generation of anxiety-like behavior at acute time points*

**Fig. 3:**
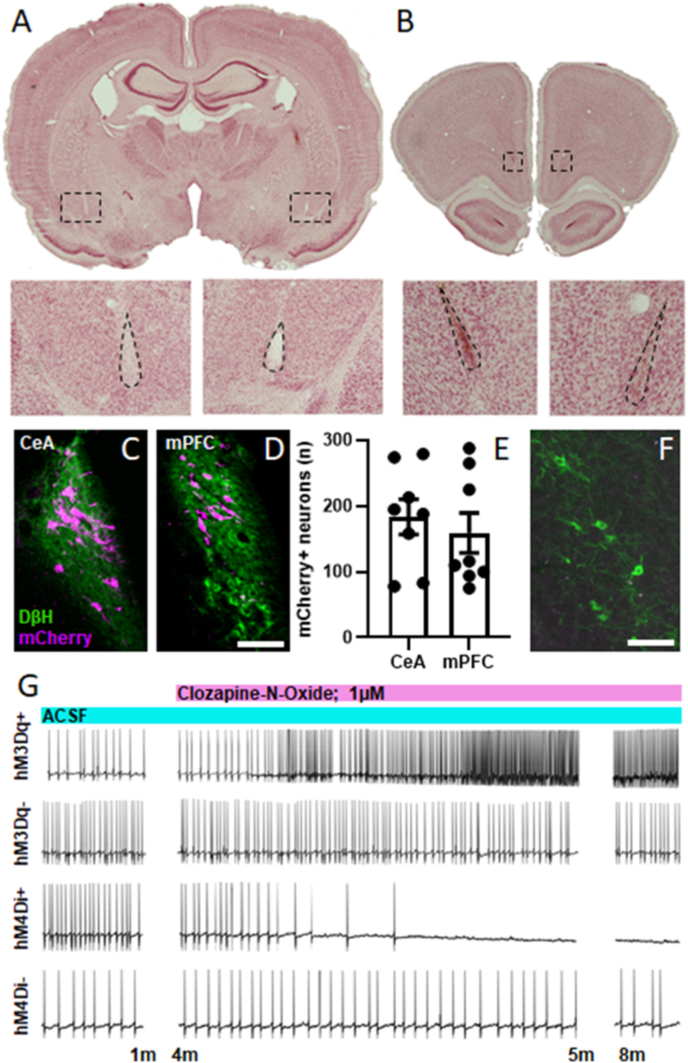
**A retrograde viral-genetic technique permits experimental manipulation of LC neurons innervating specific terminal fields.** Rats received bilateral injections of CAV2-PRS-CreV5 in CeA (n = 8) or mPFC (n = 8) and bilateral injections of Cre-inducible AAV-DIO-hM3Dq-mCherry or AAV-DIO-hM4Di-mCherry in LC. Representative images of injection sites in CeA and mPFC are shown in A&B, respectively. Boxes in low power images show the locations of high power images underneath. Areas enclosed in dotted lines in high power images show cannula tracks in CeA and evidence of minor glial scarring in mPFC that resulted from viral injections. Representative images of transduced neurons projecting to CeA and mPFC are shown in C&D, respectively. (E) These manipulations result in similar numbers of transduced neurons within each population. Noradrenergic neurons outside of LC were not found to express mCherry after these injections. A representative image of noradrenergic neurons in A5 is shown in F. Whole-cell recordings show that an hM3Dq + neuron increased its firing rate and an hM4Di + neuron was silenced in response to bath application of 1 μM CNO, respectively. Neurons lacking hM3Dq and hM4Di were insensitive to CNO (G). Scale bars in C, D&F = 100 μm.

Based on the fact that LC cells innervating CeA and mPFC underwent opposing adaptations in response to stressor exposure, we hypothesized that each contributes to anxiety-like behavior in a distinct way. Rats underwent a surgical procedure to inject the retrogradely transported NE-selective viral construct CAV2-PRS-CreV5 into either mPFC or CeA and Cre-inducible AAV2-hSyn-DIO-hM3Dq-mCherry, AAV2-hSyn-DIO-hM4Di-mCherry, or AAV2-hSyn-DIO-mCherry into LC. Representative images of Nissl-stained tissue with forebrain injection sites are shown in Fig. 3A&B. This approach permitted expression of excitatory or inhibitory DREADDs (or a control fluorescent protein) within these subsets of LC neurons (Fig. 3C, D&E). Transgene expression was not observed in adjacent noradrenergic cell bodies in A5 or A7 (Fig. 3F). To confirm that the activity of retrogradely transduced LC neurons could be manipulated by CNO, LC neurons expressing hM3Dq-mCherry or hM4Di-mCherry underwent whole-cell recordings. When exposed to 1 μM CNO in the bath, spontaneous firing was enhanced and eliminated in cells expressing hM3Dq and hM4Di, respectively. Neurons that did not express DREADDs were insensitive to CNO. Representative traces of activity from hM3Dq+, hM3Dq-, hM4Di+ and hM4Di-neurons are shown in Fig. 3G.

**Fig. 4:**
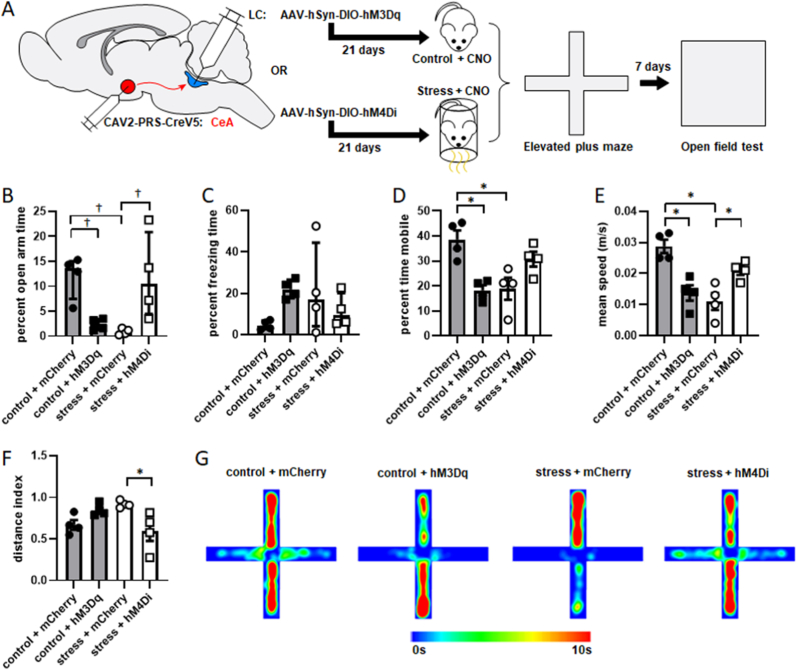
**LC→CeA neurons are acutely necessary and sufficient for anxiety-like behavior in the EPM.** The experimental timeline is shown in A. Animals underwent a surgical procedure to inject CAV2-PRS-CreV5 bilaterally in CeA and AAV-hSyn-DIO-hM3Dq-mCherry, AAV-hSyn-DIO-hM4Di-mCherry, or AAV-hSyn-DIO-mCherry bilaterally in LC. Three weeks later, all rats received an IP injection of 2.5 mg/kg CNO. Half an hour later, rats expressing hM3Dq underwent control conditions (n = 4), and rats expressing hM4Di underwent stressor exposure (n = 4). Rats expressing mCherry without a DREADD as a control were exposed to either control (n = 4) or stress (n = 4). Behavior was then assessed in the EPM and animals were returned to their home cages. One week later, behavior was assessed in the OFT. Rats were then perfused for verification of injection sites and transgene expression. Both percent open arm time and percent time freezing in the EPM were square root transformed to satisfy requirements for parametric statistical testing. (B) Rats whose LC→CeA cells expressed hM3Dq and were activated prior to control conditions spent a significantly smaller percentage of time in the open arms than control rats that expressed mCherry in LC→CeA cells. Rats whose LC→CeA cells expressed hM4Di and were inhibited prior to stressor exposure spent a significantly greater percentage of time in the open arms than stressed rats whose LC→CeA cells expressed mCherry. Stressor-exposed rats expressing mCherry in LC→CeA cells also spent significantly less time in time in the open arms than control rats expressing mCherry in LC→CeA cells (shown as median with interquartile range). (C) Freezing behavior was not significantly affected by manipulation of the LC→CeA projection (shown as median with interquartile range). Rats that had their LC→CeA projection activated prior to control conditions spent a significantly smaller percentage of time mobile than rats whose LC→CeA cells were unmanipulated. Control rats whose LC→CeA cells were unmanipulated also spent a significantly greater percentage of time mobile than stressor-exposed rats whose LC→CeA cells were unmanipulated (D). Activation of the LC→CeA projection prior to control conditions also led to significantly lower speeds in the EPM as compared to rats whose LC→CeA cells were not activated prior to control. Inhibition of LC→CeA cells prior to stressor exposure caused a significant increase in average speed relative to stressed rats whose LC→CeA cells were not inhibited. In rats whose LC→CeA cells were not manipulated, stressor exposure significantly decreased average speed relative to controls (E). To determine if these effects simply reflected altered motor function rather than alterations in anxiety-like behavior, a distance index was calculated as the amount of distance traveled in the closed arms minus the distance traveled in the open arms divided by total distance traveled. A value of 1 would indicate all travel was in closed arms, while −1 would indicate all travel was in open arms. Inhibition of LC→CeA projection cells prior to stressor exposure led to a significant decrease in distance index relative to stressed rats whose LC→CeA cells were unmanipulated, suggesting increased travel in the open arms relative to the closed arms (F). Mean heat maps for activity in the EPM are shown in G. *: p < 0.05. †: p < 0.05 after square root transformation.

To determine how these distinct LC output pathways contribute to anxiety-like behavior, each was activated 30 min prior to control condition or inhibited 30 min prior to stressor exposure (in each case with by a 2.5 mg/kg IP injection of CNO). The same manipulations were applied to both pathways to determine if the same condition within each produces a distinct behavioral response. Rats injected with AAV2-hSyn-DIO-mCherry as a vector/fluorophore control also received 2.5 mg/kg CNO IP 30 min prior to control conditions or stressor exposure to account for the effect of CNO injection (Fig. 4A). In these studies, a priori comparisons were made to determine if activation of either pathway prior to control conditions, or inhibition of either pathway during stressor exposure produced a different result than control or stress conditions on their own, rather than performing all possible comparisons. Additionally, because the goal was to determine if altering the activity of each pathway produced an effect different from its baseline activity, no statistical comparisons were drawn between activation or inhibition of one pathway with activation or inhibition of the other. This led to a one-way ANOVA with four distinct conditions for each pathway: control + mCherry, stress + mCherry, control + hM3Dq, and stress + hM4Di. Planned comparisons were performed with Bonferroni corrections applied.

A one-way ANOVA revealed a significant effect of LC→CeA pathway manipulation on percent open arm time in the EPM (F [3,12] = 10.63, p = 0.0011; Fig. 4B). Planned comparisons revealed activation of the LC→CeA pathway significantly decreased the percentage of time that control rats spent in the open arms of the EPM (p = 0.0131). Additionally, inhibition of the LC→CeA projection significantly increased the percentage of time that stressed rats spent in the open arms (p = 0.0029). Stressor exposure also significantly decreased percent open arm time in rats that expressed mCherry in the LC→CeA pathway (p = 0.0019). A one-way ANOVA did not detect a significant effect of LC→CeA pathway manipulation on percent freezing time in the EPM after square root transformation (F [3,12] = 1.997, p = 0.1194; Fig. 4C). These findings suggest that the LC→CeA projection is necessary and sufficient for the generation of anxiety-like behavior at an acute time point.

Several motor behaviors in the EPM were scored as well to determine how they were affected by pathway manipulation. A one-way ANOVA revealed a significant effect of LC→CeA pathway manipulation on percent time mobile in the EPM (F [3,12] = 8.496, p = 0.0027; Fig. 4D). Planned comparisons revealed activation of the LC→CeA projection significantly decreased the percentage of time that control rats spent mobile (p = 0.033). Stressor exposure also significantly decreased percent time mobile in rats that expressed mCherry in the LC→CeA pathway (p = 0.0042). Inhibition of the LC→CeA projection did not significantly affect percent time mobile relative in stressor-exposed rats (p = 0.0861). A one-way ANOVA revealed a significant effect of LC→CeA pathway manipulation on average speed in the EPM (F [3,12] = 12.29, p = 0.0006; Fig. 4E). Planned comparisons revealed that activation of the LC→CeA projection significantly decreased average speed in the EPM in control rats (p = 0.0016). Inhibition of the LC→CeA pathway also significantly increased average speed in stressor-exposed rats (p = 0.0269). Stressor exposure also significantly decreased average speed in rats that expressed mCherry in the LC→CeA pathway (p = 0.0004).

Because both measures of anxiety-like behavior (percent time in open arms and percent time freezing) and measures of motor function (percent time mobile and average speed) differed as a result of these manipulations, it is difficult to assess if these changes are affective or motor in origin. We therefore reasoned that if affective state was unchanged by activation or inhibition of these pathways, then altered motor activity should be confined mostly to the closed arms. Therefore, we generated a measure of how much distance was traveled in each zone normalized to total distance traveled to control for increased or decreased motor output between groups. This distance index (Fig. 4F) was calculated as distance traveled in closed arms minus distance traveled in open arms divided by total distance traveled. A value of 1 would indicate that all travel occurred in the closed arms, a value of −1 would indicate that all travel was in the open arms, and a value of zero would indicate equal amounts of travel in both regions. Because this index considers distance in both zones as well as total distance traveled, and open spaces are aversive to rodents, it can be assumed that even with increased motor output, there should not be a drop in this index without an anxiolytic or fear-reducing effect.

A one-way ANOVA revealed a significant effect of LC→CeA pathway manipulation on distance index in the EPM (F [3,12] = 4.9, p = 0.0189; Fig. 4F). Planned comparisons revealed that activation of the LC→CeA projection did not result in significantly different distance index in control animals (p = 0.2480). However, inhibition of the LC→CeA projection significantly decreased distance index (i.e., more distance traveled in the open arms) in the stressed rats (p = 0.0168). There was no also significant effect of stressor exposure on distance index in rats that expressed mCherry in the LC→CeA pathway (p = 0.0714). Mean heat maps for activity in the EPM for each condition are shown in Fig. 4G. These findings suggest that inhibition of the LC→CeA pathway leads to increased motor behavior in the open arms of the EPM, suggesting a reduction in anxiety-like behavior.

### *3.4* *LC*→*mPFC neurons contribute to both affective and motor behaviors*

**Fig. 5:**
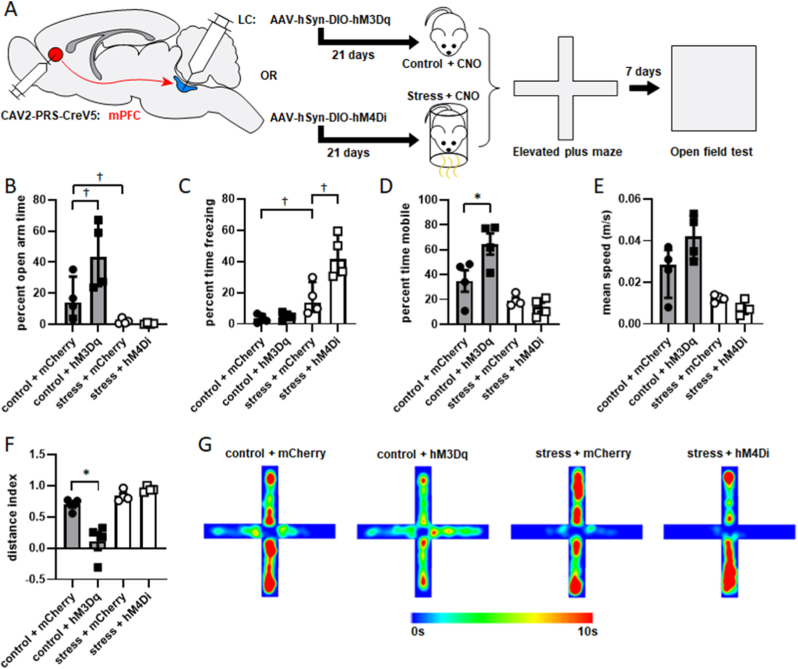
**LC→mPFC neurons promote motor hyperactivity and exploration in the elevated plus maze.** The experimental timeline is shown in A. Animals underwent a surgical procedure to inject CAV2-PRS-CreV5 bilaterally in mPFC and AAV-hSyn-DIO-hM3Dq-mCherry, AAV-hSyn-DIO-hM4Di-mCherry, or AAV-hSyn-DIO-mCherry bilaterally in LC. Three weeks later, all rats received an IP injection of 2.5 mg/kg CNO. Half an hour later, rats expressing hM3Dq underwent control conditions (n = 4), and rats expressing hM4Di underwent stressor exposure (n = 4). Rats expressing mCherry without a DREADD as a control were exposed to either control (n = 4) or stress (n = 4). Behavior was then assessed in the EPM and animals were returned to their home cages. One week later, behavior was assessed in the OFT. Rats were then perfused for verification of injection sites and transgene expression. Both percent open arm time and percent time freezing in the EPM were square root transformed to satisfy requirements for parametric statistical testing. (B) Rats whose LC→mPFC cells expressed hM3Dq and were activated prior to control conditions spent a significantly greater percentage of time in the open arms than control rats that expressed mCherry in LC→mPFC cells. Stressor-exposed rats expressing mCherry in LC→mPFC cells also spent significantly less time in time in the open arms than control rats expressing mCherry in LC→mPFC cells (shown as median with interquartile range). (C) Rats whose LC→mPFC cells expressed hM4Di and were inhibited prior to stressor exposure spent a significantly greater percentage of time freezing in the EPM than stressed rats that expressed mCherry in LC→mPFC projection cells. Stressor-exposed rats expressing mCherry in LC→mPFC cells also spent significantly more time freezing than control rats expressing mCherry in LC→mPFC cells (shown as median with interquartile range). Rats that had their LC→mPFC cells activated prior to control conditions spent also significantly greater percentage of time mobile in the EPM than control rats whose LC→mPFC cells were not activated prior to control conditions (D). Average speed in the EPM was square root transformed to satisfy requirements for parametric statistical testing and was not significantly affected by manipulation of the LC→mPFC pathway (E; shown as median with interquartile range). To determine if these effects simply reflected altered motor function rather than alterations in anxiety-like behavior, a distance index was calculated as the amount of distance traveled in the closed arms minus the distance traveled in the open arms divided by total distance traveled. A value of 1 would indicate all travel was in closed arms, while −1 would indicate all travel was in open arms. Activation of LC→mPFC cells prior to control conditions led to a significant decrease in distance index relative to rats whose LC→mPFC cells were not activated, indicating increased travel in the open arms relative to closed arms (F). Mean heat maps for activity in the EPM are shown in G. *: p < 0.05. †: p < 0.05 after square root transformation.

Another group of rats underwent a surgical procedure to transduce LC→mPFC neurons with hM3Dq, hM4Di, or mCherry. hM3Dq expressing rats had the LC→mPFC pathway activated 30 min prior to control conditions, and hM4Di expressing rats had this pathway inhibited 30 min prior to stressor exposure. Rats were then tested in the EPM (Fig. 5A). Notably, 75% of the rats whose LC→mPFC projection was activated prior to control conditions fell from the open arms of the EPM after only several minutes of testing (mean ± SEM duration to fall = 129.93 ± 4.36s). These falls appeared to occur as a result of slipping rather than active jumping. Both percent open arm time and percent freezing time were square root transformed to satisfy requirements for parametric statistical testing. A one-way ANOVA revealed a significant effect of LC→mPFC pathway manipulation on percent open arm time (F [3,12] = 18.3, p < 0.0001, Fig. 5B). Planned comparisons revealed that activation of the LC→mPFC projection prior to control conditions significantly increased the percentage of time that rats spent in the open arms (p = 0.0323). Inhibition of the LC→mPFC projection prior to stressor exposure did not significantly affect the percentage of time spent in the open arms (p > 0.9999). Stressor exposure also significantly decreased the percent open arm time in rats that expressed mCherry in this pathway (p = 0.047). A one-way ANOVA revealed a significant effect of LC→mPFC pathway manipulation on percent freezing time in the EPM (F [3,12] = 18.75, p < 0.0001; Fig. 5C). Planned comparisons revealed that inhibition of the LC→mPFC projection significantly increased the percentage of time stressed rats spent freezing (p = 0.0057). Activation of the LC→mPFC projection during control conditions did not affect freezing behavior (p > 0.9999). Stressor exposure also significantly increased percent freezing time in rats that expressed mCherry in this pathway (p = 0.0248). These findings indicate that activation of LC→mPFC neurons may decrease anxiety-like behavior and/or promote motor hyperactivity in control animals, and their inhibition may increase anxiety-like behavior and/or suppress motor output in stressed animals.

Because 75% of rats in this cohort fell from the EPM, we again sought to determine if motor behavior changed as a result of LC→mPFC pathway manipulation. Therefore, we assessed percent time mobile and average speed in the EPM. Average speed was used rather than total distance because the test duration varied among rats since several fell from the maze before the test ended. Thus, total distance (m) was normalized by test duration (s) to equate to average speed (m/s). A one-way ANOVA revealed a significant effect of LC→mPFC pathway manipulation on percent time mobile (F [3,12] = 12.59, p = 0.0005; Fig. 5D). Planned comparisons revealed that activation of the LC→mPFC pathway significantly increased the percentage of time that control rats spent mobile (p = 0.0203). Inhibition of the LC→mPFC pathway did not significantly affect percent time mobile in stressor-exposed rats. There was also no difference between control rats and stressed rats that expressed mCherry in this pathway (p = 0.3294). Mean speed in the EPM was square root transformed to satisfy requirements for parametric statistical testing and a one-way ANOVA revealed a significant effect of LC→mPFC pathway manipulation on this measure, (F [3,12] = 13.1, p = 0.0004; Fig. 5E), however, none of the planned comparisons with Bonferroni corrections were found to be significant (p > 0.05 in all cases).

Distance index was again assessed to aid in determining if increased motor output occurred preferentially in closed arms rather than open arms. A one-way ANOVA revealed a significant effect of LC→mPFC pathway manipulation on distance index in the EPM (F [3,12] = 21.76, p < 0.0001; Fig. 5F). Planned comparisons revealed that activation of the LC→mPFC projection significantly decreased distance index (i.e., more distance traveled in the open arms) in control animals (p = 0.0006). Inhibition of the LC→mPFC projection did not significantly affect distance index in stressor-exposed animals (p > 0.9999). There was also no significant effect of stressor exposure on this index in rats that expressed mCherry in the LC→mPFC pathway (p = 0.7307). Mean heat maps for activity in the EPM for each condition are shown in Fig. 5G. These findings suggest that activation of LC→mPFC neurons in control conditions promotes motor hyperactivity in the EPM. Because the increased locomotion occurred equally between open and closed arms, it may indicated reduced anxiety-like behavior.

**Fig. 6:**
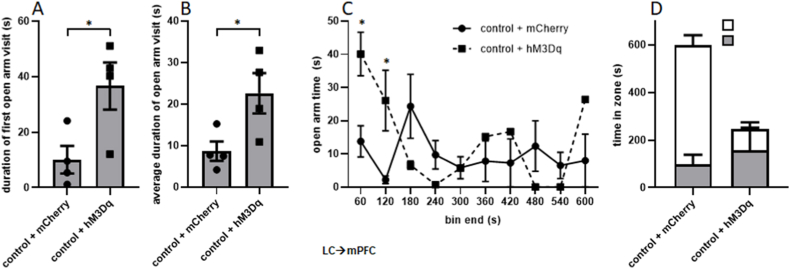
**Raw open arm time is significantly increased by activation of the LC→mPFC projection.** Because test duration varied between rats whose LC→mPFC pathway was activated as a result of falling from the EPM, several measures of open arm behavior were assessed for the time before rats fell. The duration of the first visit to the open arms, or the latency to exit the open arms after first entry, was significantly greater in rats whose LC→mPFC projection was activated (n = 4) than not activated (n = 4; A). The average duration of visits to the open arms was also significantly increased by pathway activation (B). Data were also binned in 60s increments, and rats whose LC→mPFC projection was activated spent significantly more time in the first 60s bin and the second 60s bin than rats whose pathway was not activated. No significant difference was found in the third bin when all three rats fell from the maze. No statistical comparisons could be made beyond the third bin due to only one rat remaining in this group (C). The average amount of time spent in both the open and closed arms are shown as a stacked bar graph for both groups in (D).

To further explore the possibility that anxiety-like behavior was affected by activation of the LC→mPFC projection, additional analyses were performed on open arm time for the duration of the test before rats fell from the EPM. An unpaired *t*-test showed that the duration of the first visit to the open arms was significantly greater in rats whose LC→mPFC projection was activated than unactivated (t = 2.696, p = 0.0358; Fig. 6A). This metric was chosen rather than latency to first entry to the open arms because 75% of rats in each group entered the open arms prior to the start of video recording. In addition, activating the LC→mPFC projection significantly increased the average duration of visits to the open arms (calculated as total open arm time divided by number of entries to open arms; t = 2.588, p = 0.0413; Fig. 6B). Although rats whose LC→mPFC projection was activated entered the open arms significantly fewer times than rats whose LC→mPFC projection was not activated (t = 3.101, p = 0.0211; data not shown), this was due to the fact that the test duration was shorter for those animals who fell: the number of entries per minute was unaffected by LC→mPFC activation (t = 0.4464, p = 0.8146; data not shown). When EPM open arm time was separated into 60s bins, rats whose LC→mPFC projection was activated spent significantly more time in the open arms in the first 60s bin (t = 3.243, p = 0.0176) and second 60s bin (t = 2.591, p = 0.0411; Fig. 6C) than rats whose LC→mPFC projections were not activated. No significant difference was found in the third bin when the three rats fell 3.8s, 8.2s, and 18.2s after the start of the bin (t = 1.834, p = 0.1164). Statistical comparisons were not possible beyond the third bin due to only one rat remaining in this group for those times. The average amount of raw time spent in both the open and closed arms for both groups are shown as a stacked bar graph in Fig. 6D. These findings show that prior to the falls, rats whose LC→mPFC projection was spent significantly more time in the open arms than rats whose LC→mPFC projection was not activated.

### 3.5 Manipulation of LC→CeA neurons, but not LC→mPFC neurons, alters stress-induced OFT activity one week later

**Fig. 7:**
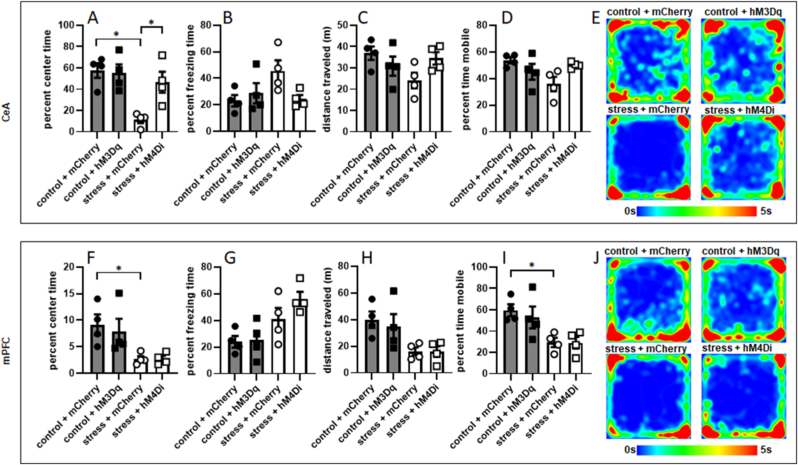
**Manipulation of LC→CeA neurons, but not LC→mPFC neurons, alters stress-induced OFT activity one week later.** (A) Rats whose LC→CeA cells were inhibited during stressor exposure (n = 4) show increased percent time in the center of the OFT one week later relative to rats that were exposed to stress and did not have their LC→CeA projection inhibited (n = 4). Additionally, stressor exposure in rats that express mCherry in LC→CeA projection cells (n = 4) significantly decreases percent time in the center of the OFT one week later relative to rats that express mCherry in LC→CeA cells and undergo control conditions (n = 4). Percent freezing time in the OFT (B), distance traveled (C) and percent time mobile (D) were not significantly affected by manipulation of LC→CeA projection cells. (E) Mean heat maps for activity in the OFT for rats whose LC→CeA pathway was manipulated. (F) Stressor exposure in rats that express mCherry in LC→mPFC projection cells significantly decreases percent time in the center of the OFT one week later relative to rats that express mCherry in LC→mPFC cells and undergo control conditions, but neither activation nor inhibition of LC→mPFC projection cells in control and stress conditions, respectively, had an effect on OFT percent center time. Percent freezing time (G) and distance traveled in the OFT (H) were not significantly affected by manipulation of LC→mPFC projection cells. (I) Stressor exposure significantly decreased percent time mobile in rats expressing mCherry in LC→mPFC cells relative to control rats expressing mCherry in this pathway. (J) Mean heat maps for activity in the OFT for rats whose LC→mPFC pathway was manipulated. *: p < 0.05.

After testing in the EPM, rats were returned to their home cages for a week. Their anxiety-like behavior was then tested in the OFT. A one-way ANOVA revealed a significant effect of LC→CeA pathway manipulation on center time in the OFT (F [3,12] = 8.051, p = 0.0033; Fig. 7A). Planned comparisons revealed that inhibition of the LC→CeA pathway during stressor exposure significantly increased time in the center of the OFT one week later (p = 0.019). Stressor exposure also significantly decreased center time in the OFT one week later in rats that expressed mCherry in LC→CeA projection neurons (p = 0.0029). Activation of the LC→CeA projection in control rats did not significantly affect OFT center time one week later (p > 0.9999). No significant effect of LC→CeA pathway manipulation was detected on freezing time in the OFT (F [3,12] = 2.879, p = 0.0801; Fig. 7B), total distance traveled in the OFT (F [3,12] = 2.372, p = 0.1216, Fig. 7C), or percent time mobile (F [3,12] = 3.396, p = 0.0537, Fig. 7D). Mean heat maps for activity in the OFT for each condition are shown in Fig. 7E.

A one-way ANOVA revealed a significant effect of LC→mPFC pathway manipulation on center time in the OFT one week later (F [3,12] = 4.406, p = 0.0262; Fig. 7F). However, planned comparisons revealed that stressor exposure significantly decreased time spent in the center of the OFT in rats that expressed mCherry in the LC→mPFC pathway (p = 0.0451), but neither activation of the LC→mPFC pathway during control conditions or inhibition of the LC→mPFC pathway during stressor exposure significantly affected OFT center time one week later (p > 0.9999 in both cases). This indicates that this effect was driven by the effect of stressor exposure on rats whose pathway was unmanipulated. A significant effect of LC→mPFC pathway manipulation was also observed on freezing time in the OFT (F [3,12] = 5.303, p = 0.0147). However, planned comparisons did not reveal any significant differences between groups (p > 0.05 in all cases; Fig. 7G). Similarly, a one-way ANOVA revealed a significant effect of LC→mPFC pathway manipulation on total distance traveled in the OFT (F [3,12] = 4.006, p = 0.0345), but planned comparisons did not reveal any significant differences (p > 0.05 in all cases, Fig. 7H). A one-way ANOVA revealed a significant effect of pathway manipulation on percent time mobile in the OFT (F [3,12] = 5.365, 0.0142) and planned comparisons showed that this measure was significantly lower in stressed rats expressing mCherry in LC→mPFC cells than control rats expressing mCherry in LC→mPFC cells (p = 0.0262, Fig. 7I), indicating that this effect was driven by stressor exposure rather than pathway manipulation. Mean heat maps for activity in the OFT for each condition are shown in Fig. 7J.

## 4 Discussion

Here we have shown that distinct groups of LC neurons innervating CeA and mPFC are differentially engaged by stressor exposure, and their activity may contribute to anxiety-like behavior in distinct ways. These findings build upon a growing body of literature that shows LC is more heterogeneous than initially recognized (Chandler et al., 2019). Additionally, this is the first study to demonstrate that distinct groups of anatomically-defined LC neurons with distinct functions also undergo opposing adaptations in response to stress. This extends our earlier finding that LC cells innervating distinct terminal fields are physiologically unique under baseline conditions (Chandler et al., 2014) to show that they adapt in specific ways in response to stress.

These studies showed that the LC→CeA projection is necessary and sufficient to generate anxiety-like behavior acutely, but not persistently. Activation of this pathway during control conditions increased anxiety-like behavior similar to that of stressor-exposed rats whose pathway is unmanipulated. Conversely, LC→CeA inhibition during stress decreased anxiety-like behavior relative to stressed rats whose pathway was unmanipulated and instead produced behavior similar to control rats. These findings support others that show that the LC projection to basolateral amygdala is critical in both fear conditioning and unconditioned negative affect (Tanaka et al., 2000; McGaugh 2004; McCall et al., 2017; Uematsu et al., 2017; Giustino and Maren 2018). Because this pathway underwent physiological adaptations in response to stress that render it more active, it may contribute to the persistent anxiety-like behavioral phenotype that we have previously reported (Borodovitsyna et al., 2018).

Our findings for the LC→CeA projection are in direct contrast to those in the LC→mPFC pathway. Whereas LC→CeA neurons became more active and depolarized in response to stressor exposure, LC→mPFC projection neurons became less so, as well as less excitable. In agreement with prior reports of LC membrane properties, 16 of 17 neurons from control animals showed spontaneous activity (Williams et al., 1984; Jedema and Grace 2004). Notably though, half of the recorded LC→mPFC neurons from stressed rats showed no spontaneous activity, and those that did showed reduced firing rates. Additionally, identical conditions for each pathway produced opposing changes in EPM behavior. Specifically, activation of the LC→CeA and LC→mPFC pathways during control conditions decreased and increased percent time in the open arms, respectively. Conversely, inhibition of the LC→CeA and LC→mPFC projections increased open arm time and increased freezing time, respectively. However, because 75% of rats whose LC→mPFC projection was activated fell from the EPM, it is difficult to assess if these effects were the result of altered motor function or affect. Although activation of this pathway increased overall percent open arm time, total time prior to falling, and the duration of the first and average visits to the open arms, it is difficult to conclude that these effects were strictly affective in origin without any effect from changes in motor circuits. It is possible that increased open arm time may be reflective of simple motor hyperactivity, rather than a lack of fear of open spaces. Future studies are therefore needed to help clarify the behavioral role of the LC→mPFC circuit. Specific assays of motor function and additional tests for anxiety-like behavior would help disentangle the confounds between motor behavior and anxiety-like behavior. For example, a lack of an effect of pathway manipulation on motor function in a locomotor or rotarod test coupled with changes in novelty-induced suppression of feeding or marble burying tasks would more strongly point to a role for the LC→mPFC pathway in anxiety-like behavior. Additionally, it is important to note that all animals in this study were adolescent males. The LC, and its response to stress, are known to be sexually dimorphic (Bangasser et al. 2010, 2011), and it undergoes a developmental trajectory that renders it less active with age (Olpe and Steinmann 1982). Furthermore, male and female rats have been shown to have distinct behavioral phenotypes and coping strategies in response to stress in various assays of anxiety-like behavior (Beck and Luine 2002; Bowman et al., 2009; Luine et al., 2017). Therefore, additional studies are also needed to resolve whether or not the stress-induced adaptations that we have identified here also occur in females, and in rats of different ages.

Some of our findings were unexpected: numerous studies have shown that the LC→mPFC circuit is activated in response, and facilitates the generation of a behavioral response, to stress (Arnsten 2000; Hirschberg et al., 2017; Giustino and Maren 2018). One potential explanation for this discrepancy is the degree to which hM3Dq activates LC neurons. Activation of LC→mPFC neurons with a different excitatory chemogenetic receptor, PSAM, which promotes aversion, increases the tonic firing rate of transduced neurons *in vivo* by a factor of ten (Hirschberg et al., 2017). Conversely, hM3Dq-mediated activation of LC neurons has been shown to result in a two to three-fold increase in tonic firing rate *in vivo* (Vazey and Aston-Jones 2014). Additionally, higher levels of LC firing are associated with release of neuropeptide co-transmitters such as galanin along with NE (Hokfelt et al., 2018). Because of the well-established role for LC-derived galanin in anxiety-like behavior (Holmes and Picciotto 2006; Sciolino et al., 2012; Weinshenker and Holmes 2016), robust PSAM-induced firing of LC neurons that produces aversion (Hirschberg et al., 2017) may be related to co-release of galanin or other co-transmitters in this circuit. Another possibility is that stress-induced NE release in mPFC aids in generating a resilient behavioral response. Stressors that are sufficiently potent to produce persistent anxiety-like behavior, however, may do so in part by suppressing the function of this circuit, and potentiating the LC→CeA projection. Reduced NE release in mPFC and increased release in CeA, then, may contribute to stress susceptibility. A similar organization of function exists in the serotonergic projection to frontal cortex and amygdala (Ren et al., 2018). Interestingly, NE excites both mPFC (Grzelka et al., 2017) and CeA neurons (Silberman and Winder 2013). However, stimulation of mPFC decreases CeA output (Quirk et al., 2003). Thus, suppression of LC→mPFC and potentiation of LC→CeA neurons by stressor exposure may reduce prefrontal inhibition of CeA, shifting the balance between these two regions towards CeA to promote persistent anxiety-like behavior.

While we found clear acute behavioral effects of pathway manipulation, one week later, there was little change. Activation of the LC→mPFC pathway in control rats did not increase exploration of the OFT one week later. Likewise, inhibition of this pathway did not increase freezing in stressed rats one week later. The acute changes may have been due to DREADD-induced changes in LC membrane potential. Because hM3Dq activates neurons through a phospholipase C and potassium channel-dependent depolarization (Alexander et al., 2009), its effects are likely to be transient. However, during stressor exposure, corticotropin releasing factor (CRF) is released onto LC, where it interacts with a Gs-coupled receptor that depolarizes neurons but also activates regulators of transcription to alter gene expression (Cibelli et al., 2001; Serova et al., 2019). Therefore, CRF release during stress may contribute to the chronic stress-induced physiological and behavioral adaptations we reported. Interestingly, the one behavior that did change persistently was that stressed rats whose LC→CeA projection was inhibited with hM4Di spent more time in the center of the OFT one week later than stressed rats whose LC→CeA neurons were not inhibited. One potential explanation for this finding is that Gi-signaling associated with hM4Di activation may have interfered with stress-induced Gs-signaling that contributes to long-term adaptation by LC neurons that contribute to persistent anxiety-like behavior (Navarro et al., 2018). Therefore, activating these circuits with a Gs-coupled DREADD in the absence of a stressor may also produce persistent effects on behavior. However, the general lack of long-term effects of DREADD-mediated activation and inhibition of LC→CeA neurons suggests that although this population is necessary and sufficient for the generation of anxiety-like behavior acutely, it is not so in the long term.

An important question that arises from these experiments is how opposing adaptations by LC neurons innervating CeA and mPFC occur. Input resistance was unaffected by stressor exposure in LC→CeA cells, but decreased in LC→mPFC cells. This may indicate increased potassium conductance in this group of cells. It has been shown that in LC, CRF, which decreases potassium conductance, also increases input resistance (Jedema and Grace 2004). Relatedly, LC spontaneous firing is mediated through PKA/cAMP signaling (Alreja and Aghajanian 1995) which decreases potassium conductance through inhibition of GIRK channels (Jedema and Grace 2004). It has also been shown that L-type, T-type and potassium-dependent calcium channels reduce LC spontaneous firing rate by increasing AHP amplitude (Matschke et al. 2015, 2018). Spontaneous firing rate was increased in LC→CeA neurons, and AHP and spontaneous firing rate were increased and decreased, respectively, in LC→mPFC neurons following stress. Therefore, differential changes in calcium and potassium channel function may occur in these two groups of cells, perhaps through alterations in PKA/cAMP-dependent signaling. Intrinsic differences in receptor signaling between these two populations may account for these changes: if a common afferent releases the same transmitter such as CRF onto both groups during stressor exposure, it is possible that the same receptor may couple to distinct intracellular signaling cascades that have opposing cellular effects between the two populations (Valentino and Bangasser 2016). Alternatively, different adaptations may result from innervation of these subsets of neurons by distinct afferents. During stressor exposure, release of distinct transmitters from these afferents may promote unique cellular adaptations by each group of cells. Evidence suggests that some LC cells innervating distinct terminals are themselves innervated by distinct afferents (Schwarz et al., 2015).

Collectively, these new observations add to a growing body of literature which shows that LC comprises anatomically and functionally distinct groups of cells that contribute to distinct aspects of behavior. Specific LC circuits have been shown to contribute to behavioral flexibility and exploration (Cope et al., 2019), feeding (Sciolino et al., 2019), spinal nociception (Hirschberg et al., 2017), and fear conditioning (Uematsu et al., 2017). While a role for the LC projection to basolateral amygdala in mediating negative affect has been reported (McCall et al., 2017), this is the first study to show a similar role for the LC→CeA projection. Importantly, these studies are the first to show that LC cells innervating distinct terminals undergo opposing adaptations in response to stress which may contribute to anxiety-like behavior. Stress-induced enhancement of LC output to CeA, and suppression of output to mPFC may alter how these regions interact to determine behavioral state. Therefore, LC sub-circuits represent potential sites of dysfunction which may contribute to the pathophysiology of disordered affect. Identifying the sources of biological variation between subsets of LC neurons innervating distinct terminal fields is therefore an important step in understanding how LC contributes to disease.

## Funding

This work was supported by 10.13039/100010269Wellcome Trust gr088373 to AEP and National Institutes of Mental Health R56MH121918 to DJC.

## CRediT authorship contribution statement

**Olga Borodovitsyna:** Investigation, Project administration, Supervision, Validation, Writing - original draft, Writing - review & editing. **Brenna C. Duffy:** Investigation, Visualization, Project administration, Writing - review & editing. **Anthony E. Pickering:** Resources, Validation, Funding acquisition. **Daniel J. Chandler:** Conceptualization, Data curation, Formal analysis, Funding acquisition, Investigation, Methodology, Project administration, Resources, Supervision, Validation, Visualization, Writing - original draft, Writing - review & editing.

## Declaration of competing interest

None.
